# Editorial

**Published:** 1901-06-15

**Authors:** 


### EDITORIAL.

The Tri-State Meeting, as advertised, proved to be one of the most largely attended dental meetings ever held in this country; with the possible exception of the World's Dental Congress in 1893, it was the largest. It was unique not alone in its conception, but in some respects, no dental conventions we have ever attended can surpass this in the general interest and the value of the contributions to all departments of the profession. Over seven hundred dentists were in attendance at this meeting. In order that we might not miss anything of the promised program we arrived at Indianapolis the evening of the day preceding the convention, only to find that many others were similarly forehanded. After a good night of rest, we spent the early morning hours in viewing the city from the top of the magnificent Soldiers' and Sailors' Monument. This is certainly the most beautiful monument of its kind that we ever saw. It is built of granite and iron, is nearly two hundred feet high, and is graceful in outline. It cost nearly $500,000, and is so substantially constructed that it will stand for centuries. From the top of the monument, reached by an elevator, we had a good view of the city and its environs in every direction. The city is built on a comparatively level stretch of ground, with four avenues diverging from Monument Place at acute angles to the other streets, which are rectangular to the points of the compass. The streets are nicely paved, mostly with asphaltum, and well protected with maple and elm shade trees. A very good system of street cars adds much to the convenience of sight-seeing. The city is clean and well supplied with the hardest water we ever encountered. By the last census it shows a population of nearly 170,000, an increase of 50 percent in ten years. It has good hotel accommodations, at least we so found them. It must be the most orderly city in the world. We only saw two policemen while there, and one of these was kept busy directing the street cars at a prominent corner and the other ran the elevator in the monument. We suspect the local committee had induced the mayor to send them off on a vacation that week. The meeting place was the German House, which is a club-house containing most admirable accommodations. There was a large hall which would accommodate more than a thousand, a small amphitheater which would seat five or six hundred, and plenty of space for the clinics and exhibits, and a large open air garden for the promenade and band concert. The arrangements for taking care of the guests were all that could be desired. At the entrance was a booth containing clerks to register each visitor, receipt for railway tickets and give such information as might be needed to make every one feel at home. A neat button was given to every one registering, which served as a passport to all meetings and every hospitality the citizen could offer ; and nobody seemed to begrudge anything they had.

While waiting for the opening of the first session, we spent the time inspecting the exhibits. We never got a chance to do this part of our visit justice, as there were nearly forty exhibitors and some of these very elaborate. The S. S. White Dental Manfg. Co. had a fine display, filling a large room and requiring six men to look after it. It is said to have cost more than a thousand dollars to transport and install. It was systematically arranged and was full of interest and of great educational value. There were many other displays of interest and value. We do not remember to have ever seen so complete an exhibit of dental appliances, and we are sure that this feature was not the least valuable part of the meeting. It may have proved the most expensive part of the program to some, as there certainly was a great temptation to bring home some of those attractive instruments and appliances which are designed to facilitate our too tedious operations.

At fifteen minutes after ten o'clock, the advertised hour, Dr. Geo. E. Hunt, the President of the Indiana Dental Society, mounted the platform of the great hall and called the convention to order. In most cordial language we were made welcome to the State and to everything which the local Committee of Arrangements had provided for our comfort and the convenience of the meeting. He also read a letter from the mayor of the city, extending its hospitalities. The hospitality was fittingly accepted by President Fowler, of Michigan, and President Harvey, of Ohio. The Executive Committee then reported the program, and the convention was declared duly in session and its conduct placed in the hands of the presidents of the three State societies as presiding officers. From this time on there was no loss of time. The meetings were called promptly on time, and the time was fully occupied until the last paper was read and its discussion finished on Thursday afternoon. No time was wasted on ceremony or for lack of a subject to discuss. Every paper announced on the program was read and in the order as printed. When the discussion on the last paper was finished the presiding officer took up his hat and left the hall, without pronouncing a benediction ; the audience followed, and the third meeting of Ohio, Indiana and Michigan dentists was over.

---

THE question as to the future conventions of the three States of Ohio, Indiana and Michigan was frequently discussed by the delegates at Indianapolis. It was generally conceded that they should be continued. Kentucky and Illinois were largely represented and would be glad to accept an invitation to join in future convocations. This would no doubt add to the numbers present and also to the value of the contributions. It would require extraordinary facilities for handling a body so large, and it might be considerable of a task to entertain it, although there was no sense of overcrowding experienced at the last meeting except in the program. The papers were so good and on such practical themes that every one present wanted to discuss them. This should be changed in future meetings so that but one paper may be read at each session instead of two.

---

THE question that was frequently asked at the recent Tri-State Meeting was: What is it that makes these meetings so attractive? We confess that we do not know, unless it is that they are so democratic in character. They are entirely free from opportunities for self-aggrandizement. There are no offices to fill, no salaries to draw, and not much chance for any one to become a social or professional hero. The program is made up by unseen hands and is discreetly carried out without any "ring-master." There is no technical business to be enacted. Everybody works, and apparently without being told to do so. It seems that every one expects the best meeting he ever attended and does what he can to realize his expectations.

A Pleasant incident of the meeting was the presentation to Dr. G. E. Hunt by the members present of a handsome mahogany Davenport sofa, in appreciation of his indefatigable efforts to provide for every one's comfort. The exhibitors presented to Dr. Kaylor a beautiful gold watch-charm set with diamonds.

A Memorial Tablet to Dr. Horace H. Hayden and Chapin A. Harris has been placed in the Baltimore Dental College and in the University of Maryland. This tablet is to commemorate the work of these two leaders of thought in the early days of the dental profession in this country.

The Mahoning Valley Dental Society has been organized at Youngstown, Ohio. Dr. F. S. Whitslar was elected president and Dr. Young secretary. Its next meeting will be held at Warren, Ohio, in October. It is to meet twice each year. With such capable enthusiasts as Drs. Whitslar and Corodyn Palmer as members, this organization ought to be a success.

The Alumni Association of the Louisville College of Dentistry held its annual meeting May 7, 1901. The exercises consisted of a brief literary program and an excellent line of practical clinics.

The Medical Department of Pennsylvania University is to have a fine new medical laboratory, to cost nearly $500,000. It is very encouraging to note the wonderful facilities constantly being added for facilitating the study of medical science.

---

The Alumni Association of the Ohio Dental College has erected in the College Hall a marble tablet commemorating the valued services to that school of Dr. George W. Keely, a most fit and proper proceeding. Dr. Keely gave much of his time and strength to fostering this school and the profession in Southwestern Ohio.

